# Hospital Support a Public Duty

**Published:** 1886-11-27

**Authors:** 


### Hospital Support a Public Duty.

By The Most Noble The Marquis of Salisbury, K.G.

*The General Depression of the Times.*

The apology, the defence which we have for an universal movement in favour of the hospitals is furnished by the peculiar circumstances of the times in which we are living. I do not refer only to the great state of depression of trade and industry in London, though that is an argument which has often been alluded to, and is one of the most cogent. It is a reason that applies more especially to the East of London, so thickly populated, so industrious, so full of energy and labour, but where the accumulated wealth of the metropolis is not to be found to the same extent as elsewhere. And the consequence is that, when depression of trade comes, it affects a vast number of purses, it affects all those small subscriptions upon which the support of the hospitals is dependent in the East of London. Men feel that, struggling as they are against the evil and sinister tendency of the times, they may be well excused if they diminish the offerings which they formerly brought forward gladly. As the difficulty of subscribing increases, the misery is increasing also. That very want of enterprise, that very depression which affects all, increases the wants of the poor; it spreads the depressing and fatal influence of insufficient nourishment; and the evils of illness or disease which might claim assistance from the hospitals at another time are made worse. It is less easy to cure the spread of the evil over a larger area because of the commercial suffering against which such vast multitudes of our poorer brethren are struggling. That is one of the arguments that should urge all to special exertion at this time. It is one that I hope is transitory. But we must make an effort to get over it without allowing these magnificent institutions to fall into arrear or diminish in utility. It is the feeling that further action is necessary on behalf of the Hospitals which has led me to accede to the request that I should restate the views I expressed at the meeting at Stratford in June last.

*A small Pecuniary Effort needed.*

I confess that when I consider the tremendous interests which we all wish to promote; when I consider the comparatively small pecuniary effort that is required in order to stem so much suffering, to remedy so much misery; and when I look outside and think how much energy, how much money, is being spent on other matters, I cannot but have melancholy feeling about it. It has been well said,

> "How small of all that human hearts endure
> That part which laws or kings can cause or cure."

And the contrast between the difficulty of sustaining these admirable institutions, the effort that it requires to collect the small sum that is necessary, with the tremendous energy that is characterising our movements out of doors, makes us feel that there is, perhaps, a little disproportion in our enthusiasm—that we care much for that which is temporary and small, but little for that which is more important than any political interests—viz., the vast amount of human suffering contained in this tremendous population.

*Hospitals suffer on account of their Excellence.*

Perhaps it is that the hospitals suffer because no one disputes their excellence. If they were an interest about whose value there was any controversy they would have their assailants and defenders; and their defenders and assailants would be very keen, and parties would be formed, and there would be leaders and programmes, and, no doubt, great political efforts, and large sums of money collected, and great struggling and political life on both sides. But the hospitals have the misfortune of being so undoubtedly good that nobody will venture to attack them; and therefore the defence of them is comparatively lukewarm and rare. I do not know whether we shall be able to persuade anybody to act the part of what is called elsewhere "the devil's advocate," and give the hospitals a chance of vigorous controversial existence by attacking them thoroughly well; but I am sure it would be very good for them. Not that there would be any defects discovered, for I do not believe there are any; but I believe that it would call attention to them; and in England no duty is thoroughly well performed unless a little fighting is involved in it.

*A Priority of Claim.*

But, seriously, I would urge upon all that this claim of the hospitals is prior to any other claim that can be made. It is the only philanthropic kind of charity against which the sternest and most rigid professor of political economy has never ventured to raise his voice. If you relieve the misery that you see in the streets you may be said to be encouraging beggary and pauperisation. You are always exposed to the risk, if you try to relieve suffering, of seeming to discourage the individual effort by which that suffering should have been attacked. But the sufferings which the hospitals relieve are those which no foresight can prevent, and no industry or energy can cure entirely of themselves. It is a kind of work which has no drawback; a kind of charity which leaves no evils behind it; and it is precisely, perhaps, for that reason that it requires more effort to advocate it than is the case with other charities. No doubt these are times of depression. Everybody has less to give than he had before, and we feel that especially when we have to rely on the small contributions which are diminished by distress.

Everybody must give Something.

But everybody, though giving less, must give something. A certain proportion of his income would be necessary in order to remedy the sufferings of those less fortunate than himself; and let me urge my readers rather to put other objects of philanthropy behind; rather, if so it must be, to cease from gifts elsewhere, than to allow this splendid machinery for succouring the masses of human suffering to run any risk of having its utility abridged, or of being thrown upon the dangerous, and insecure, and delusive support of State contributions. These are evils which are worth very serious attention on our part. And, depend upon it, they are evils from which no one can hope to escape the responsibility by saying that his own position in life is low, and his own means restricted. No doubt the heavier call is upon the rich; but it is equally upon all, and this deficiency will never be filled up unless the vast masses of those whose incomes are not large will each feel the responsibility of contributing what he can to so glorious an object.

